# Internal workload in elite female football players during the whole in-season: starters vs non-starters

**DOI:** 10.5114/biolsport.2023.124849

**Published:** 2023-03-07

**Authors:** Blanca Romero-Moraleda, Jaime González-García, Esther Morencos, Verónica Giráldez-Costas, José María Moya, Rodrigo Ramirez-Campillo

**Affiliations:** 1 Department of Physical Education, Sport and Human Movement, Autonomous University of Madrid, Madrid, Spain; 2 Applied biomechanics and sports technology research group, Autonomous University of Madrid, Madrid, Spain; 3 Exercise and Sport Sciences, Faculty of Health Science, Universidad Francisco de Vitoria, 28223 Pozuelo, Spain; 4 Camilo José Cela University. Exercise Physiology Laboratory. Madrid, Spain; 5 Exercise and Rehabilitation Sciences Laboratory. School of Physical Therapy. Faculty of Rehabilitation Sciences. Universidad Andres Bello. Santiago. Chile

**Keywords:** Workload, Training, Women, Soccer, Sports

## Abstract

The aim of this study was to quantify weekly internal workload across the in-season and compare the workload variables between starter and non-starter Spanish female first league (Liga Iberdrola) football players. Twenty-six participants belonging to the same team (age, height, and mass: 25.4 ± 6.1 years, 167.4 ± 4.8 cm and 57.96 ± 6.28 kg, respectively) participated in this study. Training loads (TL) and match loads (ML) were assessed through breath-cardiovascular (RPE_breath_), leg-musculature (RPE_leg_) and cognitive (RPE_cog_) rating of perceived exertion (RPE_0–10_) for each training session and match during the in-season phase (35 weeks). Session-RPE (sRPE) was calculated by multiplying each RPE value by session duration (minutes). From these, total weekly TL (weekly TL+ML), weekly TL, weekly ML, chronic workload, acute:chronic workload ratio, training monotony, and training strain were calculated. Linear mixed models were used to assess differences for each dependent variable, with playing time (starter vs non-starter players) used as a fixed factor, and athlete, week, and team as random factors. The results showed that total weekly TL (d = 1.23–2.04), weekly ML (d = 4.65–5.31), training monotony (d = 0.48–1.66) and training strain (d = 0.24–1.82) for RPE_breath_, RPE_leg_ and RPE_cog_ were higher for starters in comparison with non-starters (p = 0.01). Coaches involved in elite female football should consider implementing differential sRPE monitoring strategies to optimize the weekly load distribution for starters and non-starters and to introduce compensatory strategies to equalise players’ total weekly load.

## INTRODUCTION

Women’s football (soccer) has changed exponentially, imposing greater match load (ML) and training load (TL) demands [1]. In terms of absolute external load, the total distance covered in a match increased from ~8.5 [2] to ~10 km [3, 4], with 1,326 to 1,641 activity pattern changes, requiring decelerations, accelerations, sprinting or jumping [2]. Elite female football players cover a relative total distance up to 104.4 m · min^−1^, of which 11.4 ± 4.3 m · min^−1^ is at high intensity (> 15 km · h^−1^), 5.1 ± 2.4 m · min^−1^ at very high intensity (≥ 18 km · h^−1^) and 2.45 ± 0.8 m · min^−1^ while sprinting (≥ 21 km · h^−1^) [5].

HIGHLIGHTS

– The monitoring of differential sRPE (i.e. RPE_breath_, RPE_leg_ and RPE_cognitive_) has shown higher internal weekly loads, match load, monotony and strain for starter compared to non-starter players.
– On MD+1 non-starter players experienced higher differential sRPE than starters due to the nature of the compensation session. However, the total weekly load was higher for starters as they presented higher TL on MD-4, MD-3, MD-2 and during the match.
– As higher internal weekly load was experienced by starters, coaches may adjust the weekly training loads for non-starters to optimize load distribution for all players.

To cope with these match demands and to provide an adequate training stimulus which optimizes performance and minimizes the risk of injuries [6, 7], the assessment of TL and ML becomes crucial. In these terms, the assessment of TL and ML through ratings of perceived effort (RPE) has proved to be a valid measure of training load due to its relationships with internal and external load measures [8]. It was also identified as a useful time and cost-effective measure to understand the internal response to workload stimuli. Session ratings of perceived exertion (sRPE) have previously been widely used as a marker of internal training load [1]. However, it has previously been suggested that a general RPE is too simplistic to identify all the subjective sensations perceived during the exercise (i.e., cardiorespiratory sensations, muscular exertion, joint and skin sensation, perception of effort, fatigue, heat or pain), leading to low-reliability measures in team sports due to the intermittent nature of their physical actions [8, 9]. To overcome these constraints, differential ratings of perceived exertion (dRPE) – which integrate the cardiorespiratory system (RPE_breath_), neuromuscular system (RPE_leg_) and the cognitive RPE (RPE_cog_) [10] – have emerged as a viable alternative for measuring internal loads.

In team sports such as rugby, basketball and American football [11–13], TL and ML are widely monitored through RPE across the microcycle and the different phases of the season, with an association between workload and injury risk. For example, Malone et al. (2017) found that Gaelic football players were at increased risk of injury with a weekly cumulative TL between ≥ 1,500 and ≤ 2,120 arbitrary units (AU), with further increases in TL or large weekly changes in TL leading to a greater increase in injury risk. However, to our knowledge, there are no published data regarding weekly internal TL and ML in highly competitive female football players across extended periods of time (whole in-season).

As playing time is considered an important factor to programme the weekly workload, coaches usually plan a lower TL for starters and a greater TL for non-starters in the training session after the match day (MD+1). Indeed, on MD+1 it is common that players who performed > 60 minutes of competition (starters) complete a recovery training session, while those with < 60 minutes of competition (non-starters) complete a compensatory session [14]. Despite the compensatory sessions, non-starter male football players performed with considerably lower TL compared to starters, contributing to a lower total weekly TL for non-starters [15]. Specifically, very large (*d* = 2.0–3.4) differences between starters and non-starters were observed at high-intensity running zones in English Premier League players [16]. In this sense, consideration of players as starters and non-starters is of key importance for optimal monitoring of total weekly load and fluctuations (spikes) in TL, thus helping to optimize the player’s physical fitness and avoid increasing injury risk. Moreover, the acute:chronic workload ratio (ACWR), calculated as the ratio between acute (accumulated workload during the last 7 days) and chronic (mean load over the previous 3 to 6 weeks) workloads, has been identified as an index of player preparedness and suggested as a valuable monitoring variable [17, 18]. Nonetheless, the validity of ACWR in some sports and populations is still under discussion [19]. Indeed, the ACWR and longitudinal description of the differential sRPE in highly competitive female football players during the in-season is under-researched.

Due to its ease of application, its low cost and its practicality for monitoring the internal training load, the aims of the current investigation were to quantify the weekly internal workload across the in-season and compare several workload variables between starter and non-starter elite female football players. Based on previous literature [15], we hypothesize significant differences in internal load between starters and non-starters.

## MATERIALS AND METHODS

To fulfil the research aim, and to identify the possible differences between starters and non-starters, differentiated sRPE monitoring of the internal load was carried out during the 2018/2019 season in an elite female football team using a descriptive-comparative design. During all weeks of the competitive period, weekly load, consisting of TL and ML, was monitored for RPE_breath_, RPE_leg_ and RPE_cog_ during field-based training.

### Subjects

Initially, 26 elite female football players belonging to the same team of the first women’s Spanish League with a mean (± SD) age, height, and body mass of 25.4 ± 6.1 years, 167.4 ± 4.8 cm and 57.96 ± 6.28 kg, respectively, with 1–14 years of elite competitive experience, participated in this study. To be eligible, players had to meet the following requirements: 1) complete the whole training session, 2) no long-term injuries (> 6 months) and 3) complete > 90% of the sessions with the first team. Players were analysed as starters (completed > 60 minutes of competition in MD; mean (± SD) = 86.12 ± 8.69) and non-starters (completed < 60 minutes of competition in MD; mean (± SD) = 25.28 ± 15.83). From the 26 participants initially enrolled in the study, seven players were removed from the study due to severe injury (n = 1), completion of < 10 training sessions (n = 1), or involvement in the second team (n = 5). 19 players, excluding goalkeepers, were included for analysis (defenders = 6; midfielders = 7; attackers = 6). During the preseason, all players were informed about the aim and procedures of the study in accordance with the Declaration of Helsinki and provided informed consent. Ethical approval was obtained from Autonomous University, Madrid. Approval number: CEI-124-2528.

### Design

**FIG. 1:**
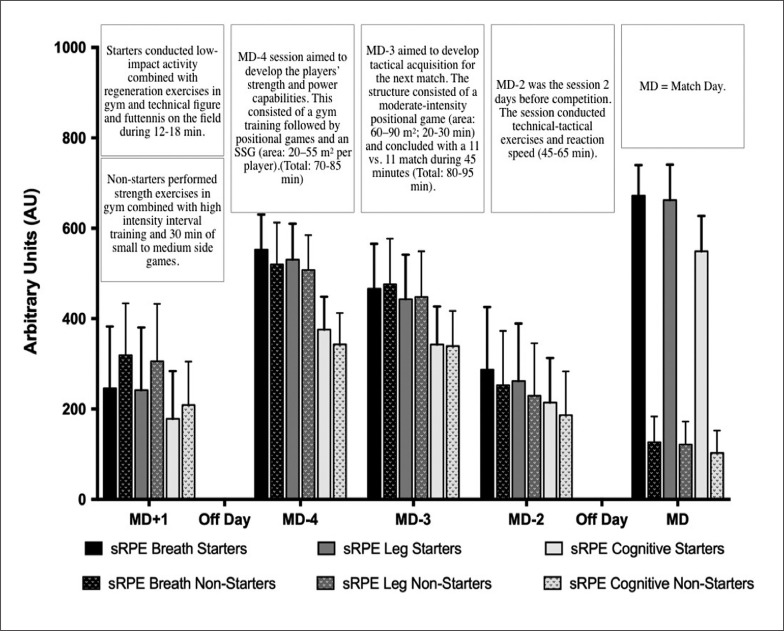
Distribution of differential sRPE and training description over a typical microcycle.

Player workload was assessed in each training session and match during the in-season (35 weeks, September 2018 to May 2019), comprising 3,197 observations during training sessions and 576 during matches. Across the in-season, players performed 30 league matches and 2 national cup (Queen’s cup) matches, without international matches. The typical weekly training and competition structure across the in-season is depicted in Figure 1, with four sessions with one match per week.

The dependent variables analysed in this study were total weekly TL, weekly TL, weekly ML, ACWR, chronic workload, training monotony, and training strain [20]. Data were compared between starters (number of cases = 2.188) and non-starters (number of cases = 1.585).

### Procedures

Internal player workload was measured through session RPE (sRPE) for each scale sRPE_breath_, sRPE_leg_ and sRPE_cog_, calculated as each RPE value multiplied by session duration (minutes), for each training session (including the recovery period between exercises) and match (excluding warm-up) [9, 21]. Players filled a questionnaire composed of three questions: i) How hard was your session on your heart/chest? (i.e., RPE_breath_), ii) How hard was your session on your legs? (i.e., RPE_leg_), iii) How difficult was your session for decision making? (RPE_cog_). Data were provided using their personal mobile phone with responses stored using cloud-based software (Google Forms, CA, United States of America). The 10-point RPE Borg scale was applied 15–30 minutes after training sessions and matches [22]. This online data collection approach has shown a stronger association with the heart rate-based training impulse (TRIMP) compared to a paper-pencil method [23], suggesting sRPE as a valid indicator of global internal load in soccer [24]. All players were familiarized with the RPE_breath_, RPE_leg_ and RPE_cog_ during six weeks of pre-season training sessions and friendly matches. The descriptions of the dependent variables for sRPEbreath, sRPE_leg_ and sRPE_cog_ (AU) are displayed in Table 1.

**TABLE 1:** Description of the dependent variables analysed

Dependent variables	Description
Total Weekly Load	Weekly TL+ Weekly ML
Weekly TL	Sum of workload from each training session in a week
Weekly ML	Sum of match workload in a week
Chronic Weekly Load	Total weekly load of the last four weeks
Acute:Chronic Workload Ratio	Total weekly load / chronic weekly load
Monotony	Mean total weekly load / SD total weekly load
Strain	Total weekly load * monotony

TL = training load; ML = match load; SD = standard deviation.

These dependent variables were calculated for sRPE_breath_, sRPE_leg_ and sRPE_cog_.

### Statistical analysis

Data are presented as mean ± standard deviation (SD) and confidence intervals (95%). Sample size was estimated using free software (G∗ Power v3.1). An a priori sample size for two independent groups revealed a minimum of 7 participants in each group given a Cohen’s d of 2.04, giving a power of 0.974. Data were analysed using factorial linear mixed modelling using the software IBM SPSS Statistics v26.0 software (IBM Corp., Armonk, NY, USA.). Variance and sphericity assumptions were checked with the Levene and Mauchly tests. Weekly changes in internal load were presented as percentages of change (%) in comparison to the previous week. Linear mixed modelling can be applied to repeated measures data from unbalanced designs, which was the case in our study since players differed in terms of the number of training sessions and matches they participated in. In this study, weekly internal load, and player group (starters vs non-starters) were treated as categorical fixed effects. The Bonferroni test was used as a post hoc test to assess where differences occurred, with Cohen’s *d* being used to calculate effect sizes. The magnitude of differences in all dependent variables between starters and non-starters was assessed using effect size (ES) statistics and was interpreted as < 0.2 = *trivial*, 0.2–0.6 = *small*, > 0.6–1.2 = *moderate*, > 1.2–2.0 = *large*, > 2.0 = *very large* [25]. Significance was set at p < 0.05.

## RESULTS

The comparison of all dependent variables between starters vs non-starters is presented in Table 2. In general, the internal loads were higher for starters than non-starters for the three differential sRPE. Total weekly load (p = 0.001; d = 1.23 to 1.44; % difference = 26.82 to 44.05%), weekly match load (p = 0.001; d = 4.58 to 5.31; % difference = 82.86 to 93.56%), monotony load (p = 0.001; d = 0.48 to 1.66; % difference = 14.33 to 44.85%) and strain (p = 0.001; d = 0.24 to 1.82; % difference = 11.74 to 66.16%) for breath, leg and cognitive showed differences between starters and non-starters.

**TABLE 2:** Mean ± standard deviation and statistical comparisons for each dependent variable for starter compared to non-starter players.

	Starter players (n = 11) Mean ± SD	Non-starter players (n = 8) Mean ± SD	95% confidence interval			
			p value	Lower	Upper	Cohen’s d
Total Weekly Load Breath (AU)	1898 ± 385	1062 ± 431	**0.01**	640	1030	2.04
Weekly Training Load Breath (AU)	1382 ± 435	1383 ± 430	0.99	-207.	205	0.00
Weekly Match Load Breath (AU)	699 ± 165	45 ± 40	**0.01**	586	722	5.31
Chronic Weekly Load Breath (AU)	1875 ± 119	1034 ± 234	**0.01**	746	935	4.52
Acute:Chronic Workload Ratio Breath	1.02 ± 0.23	1.05 ± 0.53	0.34	-0.23	0.18	-0.07
Monotony Breath (AU)	3.55 ± 1.10	2.06 ± 0.63	**0.01**	1.06	1.92	1.66
Strain Breath (AU)	6891 ± 3215	2332 ± 1484	**0.01**	3363	5752	1.82
Total Weekly Load Leg (AU)	1816 ± 377	1329 ± 413	**0.01**	298	675	1.23
Weekly Training Load Leg (AU)	1310 ± 433	1317 ± 421	0.95	-210	196	-0.02
Weekly Match Load Leg (AU)	721 ± 171	117 ± 53	**0.01**	532	675	4.65
Chronic Weekly Load Leg (AU)	1798 ± 106	1309 ± 222	**0.01**	400	578	2.81
Acute:Chronic Workload Ratio Leg	1.02 ± 0.24	1.04 ± 0.42	0.86	-0.19	0.16	-0.04
Monotony Leg (AU)	3.49 ± 1.09	2.99 ± 0.98	**0.05**	0.01	0.99	0.48
Strain Leg (AU)	4642 ± 2343	4097 ± 2127	0.31	-522	1612	0.24
Total Weekly Load Cognitive (AU)	1407 ± 306	962 ± 314	**0.01**	297	593	1.44
Weekly Training Load Cognitive (AU)	985 ± 315	950 ± 322	0.65	-117	186	0.11
Weekly Match Load Cognitive (AU)	601 ± 142	103 ± 51	**0.01**	437	557	4.58
Chronic Weekly Load Cognitive (AU)	1388 ± 119	923 ± 188	**0.01**	385	545	2.95
Acute:Chronic Workload Ratio Cognitive	1.02 ± 0.25	1.03 ± 0.41	0.90	-0.18	0.16	-0.03
Monotony Cognitive (AU)	2.23 ± 0.47	1.86 ± 0.35	**0.01**	0.18	0.58	0.91
Strain Cognitive (AU)	3189 ± 1084	1829 ± 791	**0.01**	907	1812	1.43

**FIG. 2:**
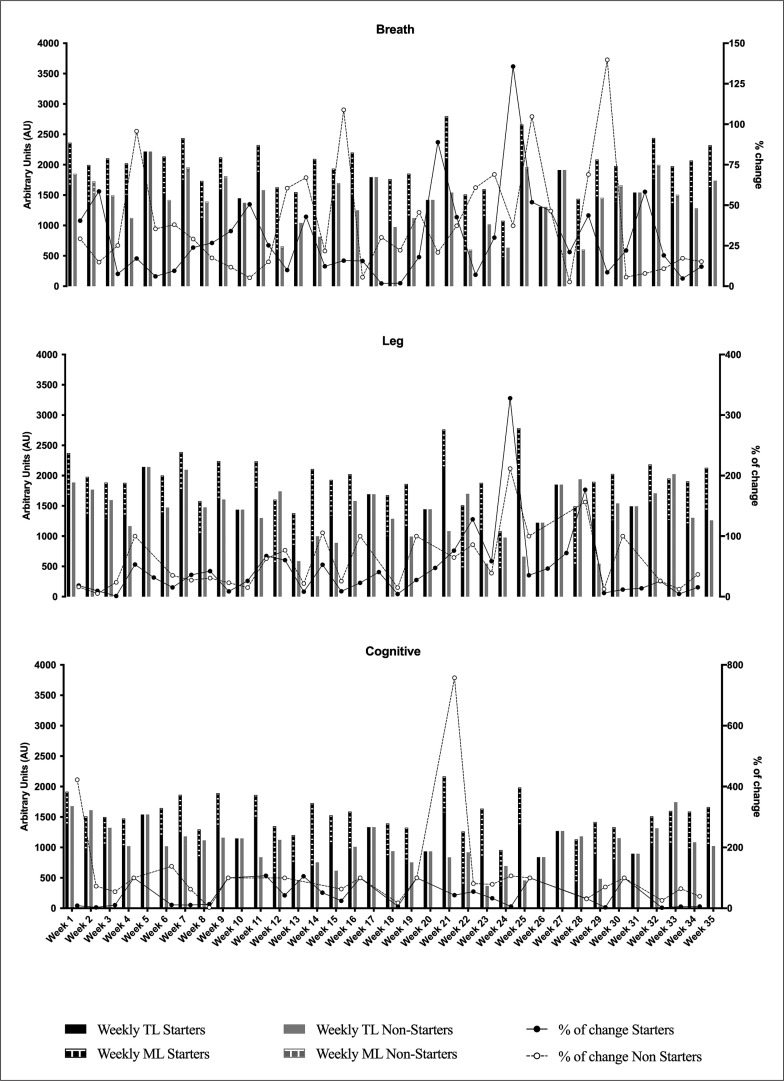
Distribution of differential sRPE for TL, ML and weekly change over the season.

The sRPE_breath_ showed an average weekly variation (as AU) of 29.75 ± 27.0% for starters and 38.96 ± 33.7% for non-starters across the in-season (Figure 2, right Y axis). The ACWR_breath_ ranged between 0.63 to 1.34 for starters and 0.58 to 2.11 for non-starters. The monotony ranged between 2.04 to 7.18 and 1.50 to 6.27 for starters and non-starters, respectively. The breath training strain was significantly higher for starters in comparison with non-starters (p = 0.01; d = 1.82; % difference = 66.16%).

A similar trend was observed for sRPE_leg_, with an average weekly variation across the season (as AU) of 29.72 ± 28.8% for starters and 40.74 ± 38.3% for non-starters (Figure 2, right Y axis). The range of ACWR_leg_ was 0.62 to 1.27 for starters and 0.59 to 2.24 for non-starters. The monotony range was 2.02 to 6.28 and 1.65 to 6.08 for starters and non-starters, respectively. Leg training strain showed no differences between starters and non-starters (p = 0.31; d = 0.24; %difference = 11.74%).

For sRPE_cog_ there was less variability than breath and leg weekly load for non-starters, showing 36.42 ± 32.8% and similar values for starters with 30.11 ± 27.6% (Figure 2, right Y axis). The range of ACWR_cog_ was 0.72 to 1.41 for starters and 0.70 to 1.64 for non-starters and monotony was to 1.50 to 3.47 and 1.20 to 2.79, respectively. The cognitive training strain was significantly higher for starters in comparison with non-starters (p = 0.01; d = 1.43; % difference = 42.65%).

## DISCUSSION

The aim of this study was to quantify the internal load in elite female football players from a first division team across the in-season, and to compare between starters and non-starters. The main finding was that starters experienced higher internal loads, including RPE_breath_, RPE_leg_ and RPE_cognitive_ for total weekly load, match load, monotony and strain.

In our study, non-starters presented higher sRPE_breath_, sRPE_leg_ and sRPE_cognitive_ on MD+1, than starters (Figure 1). Despite lower TL on MD+1 observed for starters than non-starters, there was no difference for total weekly TL, as non-starters showed lower TL in the rest of the weekly training sessions (MD-4, MD-3 and MD-2) and ML (Figure 2). These results are in agreement with Stevens et al., (2017) who reported lower TL in non-starters compared to starters, especially on MD-4, contributing to a considerably lower total weekly load for non-starters compared to starters [15]. The possible reason explaining why the non-starters experienced less TL across the different training sessions of the microcycle (except on MD+1) may be that starting players present higher RPE for the same session due to an incomplete recovery after the match [26]. However, the fact that the team line-up is indicated to starters and non-starters usually on MD-3 may reduce the motivation among non-starters, and thus their TL [27].

Despite the importance of monitoring player workload in order to optimize the training process [28–30], our study is the first longitudinal one (i.e., 35 weeks long) to quantify internal loads in highly training (first division) female football players across the in-season. Previous studies in highly trained female soccer players described the workload during a microcycle [31, 32] without comparison between starters and non-starters. The microcycle involved 7–8 days of preparation camp for an international tournament, with 4–5 training sessions, two friendly matches, and a total load of 1716 ± 603 AU [32], similar as in our study. Despite the decreases (-30.5% MD-5 vs MD-1) in workload during a typical microcycle [31], our novel results indicate meaningful differences in the internal loads between starters and non-starters. These findings may be of key importance to better assess the internal load in highly trained female soccer player, to maximize fitness and reduce injury risk.

In the current investigation, starters experienced greater (32.0 to 41.0%) internal loads compared to non-starters, with total weekly load of sRPE_breath_,sRPE_leg_, and sRPE_cognitive_ of 1,798 ± 385 AU, 1,816 ± 377 AU and 1,407 ± 306 AU among starters, and 1,062 ± 431 AU, 1,329 ± 413 AU and 962 ± 314 AU for non-starters (Table 2). Aside from the absolute values of internal load, the monitoring of weekly variations and spikes in internal loads may also be considered as relevant markers to monitor players’ performance, and avoid injury [18]. Indeed, a weekly change in workload > 15% could lead to a 21–49% increase in injury risk in team sports athletes [18]. Our results showed high weekly variations in internal loads (sRPE_breath_, sRPE_leg_, and sRPE_cognitive_) for both starters and non-starters, although larger weekly variations were noted for non-starters. Conversely, training monotony in Portuguese female players was similar between starters and non-starters (4.3 ± 0.7 vs 4.8 ± 0.8), suggesting non-significant variations in workload during the microcycle [31]. The spikes (i.e., total weekly load) for starters were observed in weeks 10, 20, 21, 24, 25, and 30, in line with those weeks that included two matches, or those weeks without a competition. For non-starters spikes for total weekly load were greater compared to starters, and occurred in weeks without competitions (weeks 10, 17, 20, 26, 27, 31) and weeks with a double match (weeks 12, 14, 22, 28). The coaching staff should consider these workload spikes to better programme higher compensatory workload increments overall for non-starters during training sessions to avoid a decrease in performance and/or increase injury risk. However, the main problem of compensatory training sessions is performing one day after a competition, causing the fatigue to occur later for non-starters; due to this, in the double match weeks it could be difficult to compensate the total weekly workload for non-starters. Despite the potential relevance of our findings in elite female football players, it should be highlighted that no data exist regarding the effect of spikes in total weekly TL on non-contact injuries in elite female football players, calling for future research in this area.

Previous studies suggested that high training monotony and workload strain levels could be associated with increases in injury risk [20, 33]. In our study, monotony and strain values were greater for starters compared to non-starters for breath, leg and cognitive scales. Higher monotony values may suggest low standard deviations of the mean with small variations within a week. Higher strain could suggest greater acute loads performed with a small within-week variation. Originally, monotony and strain workload indices were used to monitor overtraining syndrome [22]. However, several studies have associated high monotony and strain levels with increases and decreases in injury risk [33, 34]. Brink et al. (2010) analysed the workload, injuries and illness in male football players, detecting higher injury risk and illnesses when monotony was elevated. However, in another prospective longitudinal cohort study with 130 professional football players, it was noted that high monotony levels were associated with decreased injury incidence [34]. Although workload indices may help to understand the load dynamics within and between weeks, they could also be used to compare the magnitude of stimulus between players with different playing times. In our study, the leg monotony index showed greater spikes than breath and cognitive monotony indices. The starters experienced higher leg monotony levels in weeks 22, 25 and 32, while non-starters had higher levels in weeks 8 and 11. This shows how load dynamics varies greatly by playing time, the match load being one of the most important factors to optimize player performance [35]. These data suggest that it is necessary to individualize monitoring workload in female football players and propose suitable strategies to compensate the match load. Therefore, female football coaches are challenged in dosing an adequate training stimulus for their players while considering their playing time during matches.

During the 35 weeks analysed in our study, the ACWR range was higher for non-starters than starters for ACWR_breath_ (0.58–2.11 and 0.63–1.34, respectively), ACWR_leg_ (0.59–2.24 and 0.62–1.27, respectively) and ACWR_cog_ (0.70–1.63 and 0.72–1.41, respectively) ratios. While the optimal ACWR has been suggested to be between 0–85 and 1.25 to reduce injury risk in male elite football players [17], no data are available for female players, thus limiting the interpretation of our results. Indeed, female football players suffer 2 to 8 times more injuries than male players [36]. Therefore, future studies should investigate the ACWR in female football players to identify the optimal values to reduce non-contact injuries and achieve successful performance in matches, if they exist.

It is necessary to highlight the importance os using differential RPE such as RPE_breath_, sRPE_leg_, and sRPE_cognitive_ to measure internal load in elite female football players [37]. Knowing the differential RPE response can help practitioners to prescribe appropriate methods to support the specific (i.e., central or peripheral fatigue) recovery process. In our investigation, RPE_breath_ scores were substantially higher than RPE_leg_ and RPE_cognitive_ for each training session and match for starters and non-starters (Figure 1). These differences may reflect the different physiological stimuli of cardiovascular fitness and resistance training. Monitoring internal load during training and matches can aid athlete management, training prescription and decision making—ultimately facilitating player development and prescription of appropriate methods to support the recovery process.

Our data offer novel information about internal workload in highly trained female football players. However, there are some potential limitations to this study that should be acknowledged. Firstly, the current results come from a single elite female football club during the in-season period. Therefore, generalization of the current findings to teams from different competition levels (e.g., amateur), sports (e.g., basketball), or season period (e.g. preseason) should be conducted with caution. Nonetheless, the current results might be considered valid for football teams using similar weekly schedules during the in-season. Another potential limitation relates to the lack of quantification of other loads that players received in training sessions outside those conducted in the football field, such as strength and conditioning sessions in the gym. This may have caused a slight bias towards underestimation of load in strength training sessions in the gym overall, for non-starters. Future research could identify whether these reductions in total weekly training load may be due to differences in training drills, the availability of starting players or the lack of inclusion of off-field sessions. Additionally, our study did not consider playing positions and contextual factors such as match localization (i.e., home or away), final score, etc., which might potentially affect football players’ workload. Moreover, the lack of external load quantification prevents us from identifying its relationship with the internal load response.

## CONCLUSIONS

We concluded that starters experienced higher internal load, through RPE_breath_, RPE_leg_ and RPE_cog_, for total weekly load, match load, monotony and strain. Therefore, it is recommended that coaches adjust the weekly training loads for highly trained female football players, considering whether they played as starters or non-starters in the last match.

Practitioners involved in elite female football should consider implementing workload monitoring strategies to analyse the weekly load for all the players, starters and non-starters. The sRPE as a monitoring tool allows one to describe the weekly load to optimize load distribution, considering that all players should receive adequate stimuli to maintain and improve performance and to avoid injuries. Special attention should be given to non-starter players, to guarantee a balanced weekly load distribution during the in-season period. Indeed, non-starter players tended to experience a greater load on MD+1, as a compensatory mechanism for their reduced participation on MD. However, a disproportional reduction is observed toward the rest of the weekly training sessions.
